# Combined Oral Contraception and Obesity Are Strong Predictors of Low-Grade Inflammation in Healthy Individuals: Results from the Danish Blood Donor Study (DBDS)

**DOI:** 10.1371/journal.pone.0088196

**Published:** 2014-02-06

**Authors:** Cecilie J. Sørensen, Ole B. Pedersen, Mikkel S. Petersen, Erik Sørensen, Sebastian Kotzé, Lise W. Thørner, Henrik Hjalgrim, Andreas S. Rigas, Bjarne Møller, Klaus Rostgaard, Mads Riiskjær, Henrik Ullum, Christian Erikstrup

**Affiliations:** 1 Department of Clinical Immunology, Aarhus University Hospital, Aarhus, Denmark; 2 Department of Clinical Immunology, Næstved Sygehus, Næstved, Denmark; 3 Department of Clinical Immunology, Copenhagen University Hospital, Rigshospitalet, Copenhagen, Denmark; 4 Epidemiology Research, Statens Serum Institut, Copenhagen, Denmark; 5 Department of Obstetrics and Gynaecology, Aarhus University Hospital, Aarhus, Denmark; University of Leicester, United Kingdom

## Abstract

**Background:**

C-reactive protein (CRP) is a well-established marker of inflammation. The level of CRP is affected by several lifestyle factors. A slightly increased CRP level, also known as low-grade inflammation (LGI), is associated with increased risk of several diseases, especially cardiovascular disease. The aim of this study was to identify predictors of increased CRP levels in healthy individuals. We therefore assessed CRP in a large cohort of blood donors.

**Methods:**

We measured plasma CRP levels in 15,684 participants from the Danish Blood Donor Study. CRP was measured by a commercial assay. Furthermore, all participants completed a standard questionnaire on smoking status, alcohol consumption, physical activity, diet, and various body measurements. Female participants also reported the use of contraception, childbirth, and menopausal status. The relationship between LGI (defined here as a plasma CRP level between 3 mg/L and 10 mg/L) and predictors was explored by multivariable logistic regression analysis. Results were presented as odds ratios (OR) with 95% confidence intervals (CI).

**Results:**

We found LGI in a total of 1,561 (10.0%) participants. LGI was more frequent in women using combined oral contraception (OC) (29.9%) than in men (6.1%) and women not using OC (7.9%). Among premenopausal women, OC was the strongest predictor of LGI (odds ratio = 8.98, p<0.001). Additionally, body mass index (BMI) and waist circumference were positively associated with LGI.

**Conclusion:**

High BMI and abdominal obesity strongly predicted LGI among healthy individuals. However, the most striking finding was the high prevalence of LGI among premenopausal women who used combined oral contraception. Although the significance of CRP as a marker of inflammation is well known, the role of CRP in pathogenesis is still uncertain. The impact of oral contraception on CRP levels should nevertheless be considered when CRP is used in risk assessment.

## Introduction

C-reactive protein (CRP) was the first acute-phase protein to be described. It is a general marker of several pathological processes, including infection, tissue damage, cancer, and chronic inflammatory disease [1]. Plasma CRP is produced mainly by hepatocytes, predominantly under transcriptional control by the cytokine IL-6 originating from sites of pathology. CRP recognizes altered self and foreign molecules based on pattern recognition [2] and activates the classical complement pathway [1]. CRP thus acts as an opsonin, which promotes bacterial killing and induction of phagocytosis [3]. CRP has also been proposed to take part in the formation of atherothrombosis [4]. However, the clinical influence of CRP is still not completely understood [1].

CRP is a stable analyte with a plasma half-life of about 19 hours [1]. CRP levels increase rapidly up to 1000-fold during exposure to various inflammatory stimuli [5], and CRP is used routinely as a marker of infection often with a cut-off of 10 mg/L [6]. However, very low levels of CRP can be measured accurately, and it is thus possible to identify individuals with low-grade inflammation (LGI), defined as CRP measurement above 3 mg/L but below (or equal to) 10 mg/L. Previous studies suggest that most healthy individuals have CRP levels below 3 mg/L [7], but almost one third of all apparently healthy Europeans have CRP levels above this level [8].

LGI is positively associated with obesity, smoking, alcohol consumption, and high meat consumption, and is negatively associated with physical activity and fruit consumption [9]. Among women in the reproductive age, oestrogens protect against the development of atherosclerosis, but increased levels of oestrogens can induce secondary dyslipidemia (DL). Indeed, DL is sometimes observed among users of hormonal contraception, despite the lowered doses of oestrogen and progesterone in modern hormonal contraceptives. These contraceptives can furthermore affect blood pressure and coagulation factors, and use of combined oral contraception (OC) increases the level of CRP in healthy women [10]. Such observations have sparked a debate concerning the association between modern hormonal contraception and the risk of cardiovascular diseases [11].

A CRP levels above 10 mg/L is associated with increased risk of several diseases [12] including stroke, coronary heart disease [13], [14], rheumatoid arthritis [15], hypertension [16], and colorectal cancer [17]. Similarly, LGI is associated with increased risk of coronary heart disease [13] and rheumatoid arthritis [15]; indeed, CRP is widely used as a measure of LGI in cardiovascular risk assessment [4], [18]. But to rely on CRP for risk prediction, one must identify predictors of increased CRP among healthy individuals. Blood donors represent a healthy subset of the general population [19], [20] and are thus, suited for the current study. We analysed levels of CRP measurements from 15,658 participants in *The Danish Blood Donor Study* (DBDS), we determined the distribution of CRP, estimated the prevalence of LGI and identified predictors of LGI.

## Methods

### Ethics Statement

Oral and written informed consent was obtained from all participants. The study was approved by The Scientific Ethical Committee of Central Denmark (M-20090237). Additionally, the biobank and research database have been approved by the Danish Data Protection Agency (2007-58-0015).

### Participants

We studied participants from DBDS; which is an ongoing initiative established in March 2010 as a multicenter, public-health study and biobank (www.dbds.dk). In DBDS, repeat blood donors between 18 and 67 years (inclusive) are informed of the study both orally and in writing, and are then invited to participate. If the donor agrees, a four-page questionnaire is completed and routine blood samples taken. The questionnaire addresses smoking status, alcohol consumption, physical activity, diet, anthropometric measurements, and (among women) use of contraception, childbirth, and menopausal status. During the first three years, 80,000 Danish blood donors have thus far been included. Overall, fewer than 5% of the donors asked have declined the invitation to participate [21]. All participants in the present study were therefore healthy, non-pregnant, and non-lactating adults.

From 1 March to 31 December, 2010, 25,877 participants were included in DBDS. CRP was measured in plasma samples for 15,070 (91.0%) participants included between March and August and for 2,855 (30.7%) participants included between September and December. CRP was measured by a commercially available, high-sensitivity assay on an automated system (Ortho Vitros 5600, Ortho Clinical Diagnostics, Rochester, NY, USA). Blood was collected in ethylenediamine tetraacetic acid (EDTA)-containing, gel-separated tubes, centrifuged within six hours and stored at −20°C. Samples were stores for less than 12 months before CRP measurement. Measurements in thawed EDTA plasma were validated against fresh serum, the test material recommended by the manufacturer. The two measures were highly correlated (R^2^: 0.98). The CRP levels were lower in plasma (slope: 0.935). The coefficient of variation for the assay was ≤4.8%. The measuring range of the assay was 0.10 to 15.00 mg/L. A default value of 0.05 mg/L was assigned to samples below the lower limit of detection; no samples beyond the upper limit of detection were found.

We excluded 170 individuals with CRP levels above 10 mg/L. Of these, 82 were premenopausal women using OC, 38 were premenopausal women not using OC, 14 were postmenopausal women and 36 were men. Only 23 participants had CRP levels greater than 14 mg/L. In addition, we excluded participants who failed to respond to certain questionnaire items; namely current smoking (289 participants were excluded), height (167), weight (66), body mass index (BMI) (217), waist (1,232), physical activity at work (84), leisure-time physical activity (82), meat intake (112), fish consumption (237), combined oral contraception (OC) (32), progesterone-only pill (POP) (32), hormone releasing intrauterine device (IUD) (32) and birth giving (11). In total, 1,200 women and 1,045 men were thus excluded. The cumulative tobacco consumption (pack years) could not be estimated for an additional 745 participants. Because of the size of this group, these participants were excluded only from analyses in which pack-years was included as a predictor.

### Statistical Analysis

The characterization of the cohort was stratified by sex and current smoking status, and data were presented as numbers, medians with ranges, or frequency statistics.

For women, the analyses were further stratified by menopausal status as reported by the women in the questionnaire. For 102 women, menopausal status was defined according to age because information about menopausal status was missing (premenopausal: age <52 years; postmenopausal: age ≥52 years) [22]. Additionally, the premenopausal women were stratified according to the use of OC. Groups were compared by the two-sample T-test for normally distributed data, the Χ^2^ test for categorical data, and the Mann-Whitney U-test for non-normally distributed data.

The distribution of CRP was skewed to the right. The relationship between LGI (3 mg/L<CRP≤10 mg/L) and predictors was explored by multivariable logistic regression analysis stratified as explained above. Results were presented as odds ratios (OR) with 95% confidence intervals (CI).

The explored predictors included obesity (yes/no), defined as BMI greater than or equal to 30 kg/m^2^ [23], [24]; abdominal obesity (yes/no) defined in accordance with recommendations from WHO [25] as waist circumference greater than 80 cm for women and 94 cm for men; current smoking (yes/no), cumulative tobacco consumption in pack years (one pack year was defined as 20 g of tobacco every day for one year [26], where a cigarette was estimated to contain 1 g of tobacco; and a cheroot, a cigar, or a pipe 3 g of tobacco [27]); age (ten-year increments); physical activity at work (high vs. low, see definition below); leisure-time physical activity (high vs. low, see definition below); meat intake at least once per week (yes/no); and fish consumption (at least twice per week or not) [28], [29]. Additionally, childbirth (yes/no) was included as a predictor among women. For premenopausal women, OC, POP, and hormone-releasing IUD were included as predictors. The use of these methods of contraception was not independent. Therefore, each group of participants using a specific method of contraception was compared with participants not using either of OC, POP, or hormone-releasing IUD, e.g. OC users were compared to a group of POP nonusers and IUD nonusers.

Low physical activity at work was defined as mainly sitting or standing and occasionally walking. High physical activity at work was defined as walking and lifting occasionally; or heavy physical work. Low leisure–time physical activity was defined as light physical activity less than 2 hours per week or two to four times per week. High leisure–time physical activity was defined as light physical activity more than 4 hours a week, or fatiguing physical activity for at least 2 per week [30].

Statistical analysis was performed using Stata/IC 11.0 for Windows (StataCorp LP, College Station, Texas, USA). A p-value below 0.05 was considered significant.

## Results

### Characteristics of the Cohort

The characteristics of the cohort are presented in Table 1. The study comprised 15,684 individuals; 46.7% women and 53.3% men. On average, men were 2.3 years older than women (Mean age 40.8 years for men and 38.6 years for women, respectively, p<0.0001). Smokers constituted 16.4% and 15.6% of women and men, respectively. BMI was similar for smokers and non-smokers (women: p = 0.47; men: p = 0.45). Smokers were slightly older than non-smokers (women: mean difference between smokers and non-smokers.: 0.97 years, CI: 0.21–1.74 years, p = 0.01; men: mean difference 0.86 years, CI: 0.15–1.58 years, p = 0.02).

**Table 1:** Characteristics of the cohort stratified by sex and smoker status.

	Women (n = 7318)		Men (n = 8366)	
	Non-smoker	Current smoker	Non-smoker	Current smoker
**Number of participants**	6,112 (83.5%)	1,202 (16.4%)	7,061 (84.4%)	1,305 (15.6%)
**Age, years**	37.4 (27.1;47.7)	39.4 (28.2;48.7)	40.2 (30.5;49.6)	41.1 (31.1;51.5)
**Weight, kg**	67.0 (61.0;75.0)	67.0 (62.0;75.0)	84.0 (76.0;92.0)	84.0 (76.0;92.0)
**Height, cm**	169 (165;173)	169 (165;173)	182 (178;187)	182 (178;187)
**BMI, kg/m^2^**	23.4 (21.5;26.0)	23.7 (21.6;26.2)	25.1 (23.3;27.4)	25.2 (23.5;27.2)
**Obesity, BMI≥30 kg/m^2^**	562 (9.2%)	112 (9.3%)	692 (9.8%)	146 (11.2%)
**Ex-smoker**	1891 (25.8%)	–	1915 (22.9%)	–
**High physical activity, work**	1,213 (19.8%)	301 (25.0%)	1645 (23.3%)	442 (33.9%)
**High physical activity, leisure**	4,203 (68.8%)	722 (60.1%)	5,008 (70.9%)	827 (63.4%)
**Waist circumference, cm**	82.0 (76.0;90.0)	83.0 (77.0;91.0)	92.0 (86.0;99.0)	92.0 (86.0;99.0)
**Waist circumference above** **80 cm for women and 94 cm** **for men**	3358 (54.9%)	714 (59.4%)	2871 (40.7%)	522 (40.0%)
**Pack years if smoker or** **ex-smoker**	5.0 (1.2;11.3)	7.0 (1.6;16.3)	7.5 (2.4;16.8)	10.5 (2.4;22.5)
**CRP, mg/l**	0.7 (0.2;1.8)	0.7 (0.2;2.0)	0.4 (0.1;1.0)	0.6 (0.2;1.5)
CRP>3 mg/L*	863 (14.1%)	190 (15.8%)	378 (5.4%)	130 (10.0%)
**CRP>5 mg/L**	355 (5.8%)	80 (6.7%)	132 (1.9%)	52 (4.0%)
**Meat consumption (yes)**	6024 (98.6%)	1184 (98.5%)	7030 (99.6%)	1300 (99.6%)
**Fish consumption a least twice weekly**	2808 (45.9%)	514 (42.8%)	2922 (41.4%)	500 (38.3%)
**Combined oral contraceptive** **pill (yes)**	1856 (30.4%)	322 (26.8%)	–	–
**Progesterone-only pill (yes)**	127 (2.1%)	19 (1.6%)	–	–
**Hormone-releasing IUD (yes)**	580 (9.5%)	108 (9.0%)	–	–

Numbers with percentages or medians with interquartile ranges. *Note that this group also includes participants with CRP greater than 5 mg/L.

### Low-grade Inflammation was more Frequent among Women and Smokers

**Figure 1:**
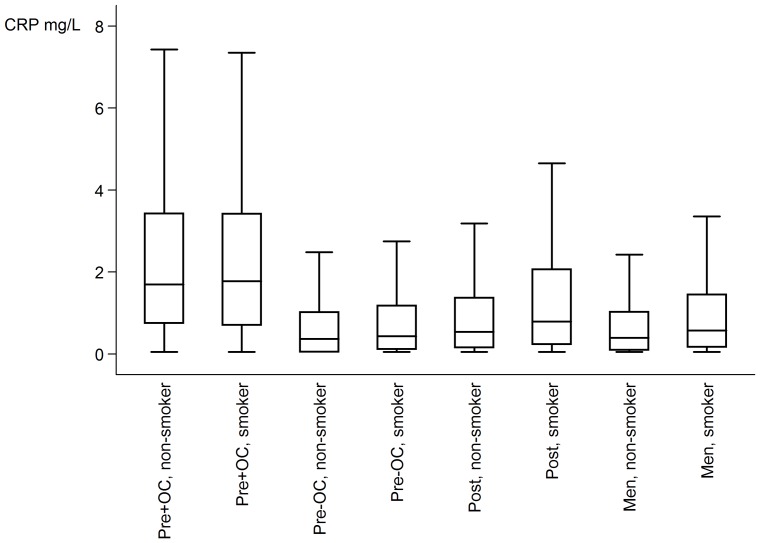
CRP levels in the cohort. Box-and-whisker plot showing the CRP levels in the four groups stratified according to smoking status. The boxes span the interquartile range with a line at the median. The whiskers represent the highest and the lowest values within 1.5 interquartile range of the nearer quartile. Due to the large size of the study, outliers are not shown. Pre+OC: premenopausal women using combined oral contraception (OC), pre-OC: premenopausal women not using OC, post: postmenopausal women.

In the total cohort, 1,561 (10.0%) had LGI. The CRP level was higher among women than among men (Mann–Whitney U test: p<0.0001) and higher for smokers than non-smokers (women: p = 0.008, men: p<0.0001) (Figure 1). Analogously, LGI was more frequent in women than in men (14.4% vs. 6.1%, p<0.001) and more frequent in current smokers than in those who had never smoked among men, but not among women (men, smokers: 10.0%, never-smokers: 4.9%, p<0.001; women, smokers: 15.8%, never-smokers: 15.1%, p = 0.56) (Table 1). Even after excluding premenopausal women using OC, more women than men had LGI (premenopausal women not using OC vs. men: p = 0.037; postmenopausal women vs. men: p<0.001). Additionally, more current smokers than ex-smokers had LGI (women, ex-smokers: 12.6%, smokers vs. ex-smokers: p = 0.011; men, ex-smokers: 6.4%, p<0.001).

### Low-grade Inflammation was Strongly Associated with the Use of Combined Oral Contraception

A total of 29.9% of premenopausal women using OC had LGI (Table 2). In comparison, only 7.1% of premenopausal women not using OC had LGI (p<0.001). The percentage of women with LGI was higher among postmenopausal women than premenopausal women not using OC (9.8% and 7.1%, respectively, p = 0.001). Because of the effect of OC, subsequent analysis was stratified for menopausal status and use of OC (Table 2). There was no difference in the prevalence of LGI between smokers and non-smokers among premenopausal women, regardless of the use of OC (OC: p = 0.83, no-OC: p = 0.38). However, for postmenopausal women, the percentage of LGI was higher among current smokers compared with non-smokers (p<0.001).

**Table 2:** Characteristics of women stratified by menopausal status and combined oral contraception (OC).

	Premenopausal women taking OC			Premenopausal women not taking OC			Postmenopausal women		
	Non-smoker	Smoker	Total	Non-smoker	Smoker	Total	Non-smoker	Smoker	Total
**Number of participants**	1,851 (85.2%)	322 (14.8%)	2,173 (100%)	3,108 (84.0%)	593 (16.0%)	3,701 (100%)	1,157 (80.1%)	287 (19.9%)	1,444 (100%)
**Age, years**	26.5 (23.6;31.9)	26.9 (23.8;32.6)	26.9 (23.6;31.9)	38.4 (30.6;44.4)	38.7(30.4;44.6)	38.4(30.5;44.5)	56.7(53.2;60.8)	55.5(51.2;59.6)	56.4 (52.7;60.5)
**BMI, kg/m^2^**	22.8 (21.1;24.9)	23.0 (21.3;25.2)	22.8 (21.1;24.9)	23.5 (21.5;26.4)	23.9(21.7;26.8)	23.6(21.6;26.4)	24.2(22.3;26.8)	24.2(22.1;26.3)	24.2 (22.2;26.8)
**CRP, mg/L**	1.7 (0.8;3.4)	1.8 (0.7;3.4)	1.7 (0.7;3.4)	0.4 (0.1;1.0)	0.4 (0.1;1.2)	0.4 (0.1;1.1)	0.5 (0.2;1.4)	0.8 (0.2;2.1)	0.6 (0.2;1.5)
**CRP>3 mg/L**	552 (29.8%)	98 (30.4%)	650 (29.9%)	215 (6.9%)	47 (7.9%)	262 (7.1%)	96 (8.3%)	45 (15.7%)	141 (9.8%)
**CRP>5 mg/L**	226 (12.2%)	41 (12.7%)	267 (12.3%)	92 (3.0%)	20 (3.4%)	112 (3.0%)	37 (3.2%)	19 (6.6%)	56 (3.9%)

Characteristics of women stratified for menopausal status and the use of combined oral contraceptives. Numbers with percentages or medians with interquartile ranges. BMI: Body Mass Index.

### Predictors of LGI

In the adjusted logistic regression analysis OC remained a powerful predictor of LGI (premenopausal women using OC: OR = 8.98, p<0.001) and subsequent analyses were stratified for sex and (for women) menopausal status and the use of OC (Table 3). For all four groups, the following predictors were included: obesity (BMI≥30 kg/m^2^), current smoking, age, and physical activity during leisure–time and at work. In addition, childbirth was included as a predictor among women. Among non–OC–users, POP and hormone–releasing IUD were predictors. When waist circumference was added to the model, BMI was excluded. Thus, either BMI or waist circumference was used as a measure of obesity. A BMI equal to or greater than 30 kg/m^2^ was positively associated with LGI in all four groups. Similarly, a waist circumference greater than 80 cm for women and 94 cm for men predicted LGI in all four groups. Consumption of meat or fish was not associated with LGI in any group.

**Table 3:** Predictors of low-grade inflammation estimated by multivariable logistic regression analysis.

	Premenopausal women taking OC (n = 2,173)		Premenopausal women not takingOC (n = 3,701)		Postmenopausal women (n = 1,471)		Men (n = 8,366)	
Outcome: Low-grade inflammation	OR	P	OR	P	OR	P	OR	p
**Obesity (BMI≥30 kg/m^2^)**	5.12 (3.65–7.80)	<10^−13^	8.00 (6.05–10.58)	<0.001	8.27 (5.51–12.43)	<0.001	3.97 (3.21–4.90)	<0.001
**Waist circumference** **(high vs. low)**	2.70 (2.22–3.28)	<0.001	3.33 (2.40–4.62)	<10^−12^	3.61 (2.11–6.18)	<10^−5^	2.11 (1.74–2.54)	<10^−14^
**Current smoking (yes/no)**	0.96 (0.73–1.25)	0.756	1.17 (0.82–1.65)	0.384	2.23 (1.47–3.40)	<0.001	1.87 (1.51–2.32)	<10^−8^
**Pack years**	1.00 (1.00–1.01)	0.246	1.00 (0.99–1.01)	0.744	1.00 (1.00–1.01)	0.482	1.00 (1.00–1.00)	0.263
**Age (10 years older)**	1.27 (1.06–1.52)	0.010	1.05 (0.88–1.26)	0.593	1.20 (0.83–1.72)	0.333	1.24 (1.15–1.33)	<10^−7^
**Physical activity, work** **(high/low)**	1.17 (0.92–1.49)	0.190	1.34 (0.99–1.83)	0.061	1.32 (0.88–1.99)	0.183	1.22 (1.00–1.49)	0.052
**Physical activity, leisure** **(high/low).**	0.72 (0.59–0.89)	0.002	0.71 (0.54–0.93)	0.013	0.66 (0.45–0.98)	0.038	0.86 (0.71–1.04)	0.116
**Meat consumption (yes/no)**	1.01 (0.44–2.35)	0.976	2.13 (0.50–9.10)	0.306	NA	NA	0.39 (0.14–1.13)	0.082
**Fish consumption at least** **twice weekly (yes/no)**	0.91 (0.75–1.11)	0.351	0.84 (0.64–1.10)	0.204	1.20 (0.82–1.75)	0.354	1.01 (0.84–1.22)	0.879
**Childbirth (yes/no)**	1.13 (0.83–1.54)	0.432	0.18 (0.56–1.07)	0.123	0.98 (0.59–1.65)	0.520	–	–
**Progesterone-only pill** **(yes/no)**	–	–	1.72 (0.87–3.38)	0.118	–	–	–	–
**Hormone releasing IUD** **(yes/no)**	–	–	0.77 (0.52–1.16)	0.216	–	–	–	–

Predictors of low-grade inflammation (CRP>3 mg/L) in multivariable logistic regression analysis. Obesity: Body Mass Index (BMI). Waist circumference, larger vs. smaller than WHO cut-offs: 80 cm for women and 94 cm for men. The analysis was stratified by sex and, among women, by menopausal status and use of combined oral contraception (OC). Predictors in all four groups included: current smoking (yes/no), age (ten-year increment), meat and fish consumption, and physical activity at work and at leisure. For the analysis of waist circumference, BMI was excluded from the analysis. The calculation of pack years was not possible for 745 participants. Thus, the analysis for pack years was made separately and includes only 14,966 participants. Among women, additionally predictors were childbirth; and among premenopausal women not using OC, the use of progesterone-only pill (POP) and hormone-releasing intrauterine devices (IUD). Among premenopausal women not taking OC, the effects of POPs were tested after excluding users of IUD. Also, the effects of IUD were tested after excluding users of POPs. For the analysis of cumulative tobacco consumption, defined as pack years, a total of 745 participants were excluded. Odds ratio (OR) including 95% confidence interval (CI), p-value (p), numbers (n), not available (NA).

Use of POPs was not significantly associated with LGI in the relevant group of premenopausal women not using OC or hormone–releasing IUD even though a trend was observed (OR = 1.72, CI = 0.87–3.38, p = 0.12). Furthermore, the use of hormone–releasing IUD was not associated with LGI among premenopausal women when compared with a group of non–OC and non–POP users (OR = 0.77, p = 0.22).

A positive association between current smoking and LGI was present for postmenopausal women and for men (women: OR = 2.23, CI = 1.47–3.40, p<0.001; men: OR = 1.87, CI = 1.51–2.23, p<10^−8^). Current smoking did not influence the risk of LGI among premenopausal women (taking OC: p = 0.76; not taking OC: p = 0.38). The cumulative tobacco consumption, defined as pack years, was not associated with LGI.

Age, as a continuous parameter, predicted LGI among men and among women taking OC (OR = 1.24 with age 10 years higher, p<10^−7^, OR = 1.27, p = 0.010, for men and OC-using women, respectively), but not among postmenopausal women and premenopausal women not taking OC (p = 0.59, p = 0.33, respectively) (Table 3).

Physical activity at work predicted LGI among men with borderline significance only (OR = 1.22, CI = 1.00–1.49, p = 0.052). Leisure-time physical activity was associated with lower risk of LGI in premenopausal women taking OC, premenopausal women not taking OC and postmenopausal women (p = 0.002, p = 0.013, p = 0.038, respectively), but not among men (p = 0.116) (Table 3).

Finally, the variation of LGI during the year was tested. No seasonal variation of LGI was found in logistic regression analysis adjusted for sex and age (p = 0.98).

## Discussion

In this large study of 15,684 healthy blood donors, we showed, that BMI and waist circumference were positively associated with LGI. Importantly, we also identified OC as a strong predictor of LGI in premenopausal women.

In the present study, more women than men had LGI. In a French study of 3,605 healthy individuals, men and women did not differ with respect to CRP [5]. In another study of 22,403 individuals, Rifai et al found no effect of gender on CRP levels when women taking hormone replacement therapy (HRT) were excluded from analysis (HRT increases CRP levels) [7]. However, in a German cohort, women had slightly, but significantly, higher CRP levels than men, possibly because estrogen affects CRP levels directly [31]. We found that OC was a very strong predictor of LGI among premenopausal women (OR = 8.98). When stratified for the use of OC, 29.9% of the users and 7.1% of the non-users had CRP above 3 mg/L. OC may affect the hepatic CRP synthesis directly without a general inflammatory response mediated by interleukin 6 (IL-6) or endothelial activation [32]. This link between OC and increased CRP is relevant for risk assessment of CVD, where an increased CRP is a risk factor [33], [34]. The use of OC may thus increase the risk of CVD, however we do not know whether CRP is a cause rather than a consequence of disease or merely an unimportant artefact of using OC [35]. Our study does not allow us to make this crucial distinction. Thus, future perspectives of this study are to investigate whether increased CRP due to OC has pathological consequences.

The method used for CRP measurement may impact results. In some studies, lower levels of CRP have been reported in EDTA samples (8–12% lower) than in serum samples [36]. We also found lower levels in thawed plasma than in serum. In the studies cited above, CRP was measured by different methods (various commercial assays), in different material (serum or plasma, with various means of anticoagulation) stored for different time periods and at different temperatures. Therefore, the comparison of absolute levels between studies may not be accurate. However, as we and others performed within-study comparisons it is unlikely that the method used has impacted conclusions of predictors of LGI.

Other smaller studies report a similar impact of OC on CRP. For example, Buchbinder et al. find that CRP levels are affected by BMI and OC use in a study of 850 blood donors. In this study, the combination, the combination of overweight and OC use results in a six-fold increase in median CRP levels among women. In addition, age and smoking are factors of lower significance [37]. In a population-based study of 2,120 adults from Finland aged 24–39 years, BMI and OC-use have the greatest influence on CRP levels. Furthermore, among men, smoking and waist circumference affect CRP levels [38]. Finally, similar results of obesity and OC use are found by Hung et al., among 4,009 Australian adults [39].

In our study, current smoking was associated with LGI only among postmenopausal women and men but not among premenopausal women. There was no association between cumulative tobacco consumption and LGI within the strata. To examine this discrepancy, we compared the cumulative tobacco consumption between premenopausal and postmenopausal women. We found a markedly higher cumulative tobacco consumption among postmenopausal women (median pack-years, premenopausal women vs. postmenopausal women: 5.0 vs. 17.5, Mann–Whitney U-test: p<0.0001). The difference in exposure leads us to speculate that postmenopausal women were more heavily exposed and thus more prone to develop LGI. Conversely, the premenopausal women might not yet have experienced the negative effects of smoking simply because they are younger.

Hormone-releasing IUD is a highly effective contraceptive widely used in Northern Europe. A study of 2,814 Finish 31-year old women found that OC-users, but not hormone-releasing IUD-users have higher serum CRP levels than non-hormonal contraception users. Furthermore, there is no statistically significant difference in CRP between POP-users and the reference group [34].

The use of OC, however, may be associated with several confounders. The main method of contraception in Europe is OC [40], and, in Denmark, approximately 68% of all women in the reproductive age use hormonal contraception at some point [41]. Moreover, OC use offers several non-contraceptive benefits, including reduced dysmenorrhea, reduced pain due to endometriosis, regular bleeding patterns, less menstrual bleeding, clearing of acne, reduced risk of ovarian epithelial cancer and endometrial cancer without increasing the risk for breast cancer [42], [43]. Overall, OC has as positive benefit–risk ratio, but the risk of venous thromboembolism is increased 3–4 fold [43]. Furthermore, the risk of venous thromboembolism varies with different types of progestogens and varying doses of oestrogen. An association between LGI and venous thromboembolism has been suggested [44]. We suggest to further investigate the impact of OC-related CRP increase on the risk of venous thromboembolism, including the association between LGI and the various generations of OC.

The frequency of non-contraceptive use of OC is not known, but it is expected that around 50% of OC users rely on OC for non-contraceptive effects. In particular, OC has a well-documented effect on dysmenorrhea and endometriosis-related pain, which affect up to 15% of pre-menopausal women [45], [46]. Although the mechanism is unclear, LGI may play a role in the pathogenesis of endometriosis [47]. Whether the high prevalence of LGI among OC users can be partly explained by a pre-existing gynecological condition remains to be elucidated.

### External and Internal Validity

In the present study a total of 10.0% of participants had LGI. This corresponds well with other studies of healthy European and American populations [8], [13]. In contrast, up to one third of all Europeans have LGI [8]. Because blood donors must fulfil strict health and lifestyle criteria, they represent a healthy subset of the general population [19], [20]; indeed, CRP values among blood donors are slightly lower compared with apparently healthy individuals from the general population [31]. A study of 468 healthy young adult blood donors found a median CRP concentration of 0.8 mg/L, a 90-percentile of 3.0 mg/L, and a 99-percentile of 10 mg/L [1]. These results compare to our findings of 0.5 mg/L (median), 3.3 mg/L (90-percentile) and 9.9 mg/L (99-percentile). It should be noted, that we did not have information on ethnicity, and we were thus not able to adjust the analyses accordingly.

In our study 170 (1.1%) participants had CRP levels greater than 10 mg/L. No participants had CRP above 15 mg/L. We suggest that CRP levels of 10–15 mg/L were due to OC, higher-than-normal LGI, or mild asymptomatic infections [1].

Blood donors also have a healthier lifestyle than the general population. Current smoking is more frequent among the general population: 24.1% of Danish men and 20.9% of Danish women are current smokers versus 15.6% and 16.4%, respectively, among blood donors. Further, 15.3% of Danish men and 13.6% of Danish women are obese compared to 10.0% and 9.2%, respectively, among Danish blood donors [48]. Our study population is thus highly selected and consequently the findings may not be directly applicable to other populations. However, the fact that the study was based on healthy individuals does allow us to eliminate the effect of overt disease on CRP.

CRP was measured on donors spread throughout the year with most during the summer season. However, no seasonal variation in the presence of LGI was found.

Apart from selection, information bias and non-response, as discussed above, our study is cross-sectional, and the causal directions between the variables studied can therefore not be established.

## Conclusion

Among premenopausal women, the strongest predictor of low-grade inflammation was the use of OC. Although CRP may not be causally involved in the development of pathology, the use of combined oral contraception should be taken into account when CRP is used in risk assessments. Further, high BMI and waist circumference were strong predictors for LGI among healthy individuals.
